# Pathologic diagnosis and molecular features of gastrointestinal stromal tumors: a mini-review

**DOI:** 10.3389/fonc.2024.1487467

**Published:** 2024-11-19

**Authors:** Younghoon Kim, Sung Hak Lee

**Affiliations:** Department of Hospital Pathology, Seoul St. Mary’s Hospital, College of Medicine, The Catholic University of Korea, Seoul, Republic of Korea

**Keywords:** gastrointestinal stromal tumor, pathology, immunohistochemistry, molecular diagnostics, targeted therapy

## Abstract

Gastrointestinal stromal tumors (GISTs) arise from the interstitial cells of Cajal, predominantly affecting the stomach and small intestine, with rare occurrences in the duodenum, rectum, and extraintestinal sites. Histologically, GISTs can present as spindle cells, epithelioid cells, or mixed morphologies, with immunohistochemical staining revealing expression of KIT (CD117) and discovered on GIST 1 (DOG1). Approximately 80% of GISTs harbor activating mutations in KIT or platelet derived growth factor receptor α (PDGFRA), which influence their clinical behavior and treatment response. SDH-deficient GISTs, associated with syndromes such as Carney triad and Carney–Stratakis syndrome, represent a distinct subgroup with unique characteristics and management challenges. The standard treatment includes surgery and imatinib for metastatic cases; however, resistance to tyrosine kinase inhibitors remains a significant hurdle, especially in pediatric and wildtype GISTs. This highlights the need for advanced therapeutic strategies and emphasizes the importance of molecular profiling in guiding treatment decisions and improving outcomes for GIST patients.

## 1 Introduction

Gastrointestinal stromal tumors (GISTs) are rare stromal tumors that originate from the interstitial cells of Cajal within the myenteric plexus of the muscularis propria (1). GISTs can occur anywhere along the gastrointestinal tract, but they are most commonly found in the stomach (60%), followed by the jejunum and ileum (30%). They are less often in the duodenum (4%–5%), rectum (4%), colon and appendix (1%–2%), and the esophagus (< 1%) (2). Extraintestinal GISTs occur in the mesentery, omentum, and retroperitoneum (1, 3, 4). These GISTs may indicate metastatic spread from an unidentified primary tumor or represent a distinct mass originating from the gastrointestinal tract. Sporadic GISTs can arise at any age but are predominantly in the sixth decade of life (average age: 60–65 years), with a subtle male predominance (5). A small percentage of GISTs affect children and teenagers. These tumors are usually KIT/PDGFRA-wildtype GISTs with succinate dehydrogenase (SDH) deficiency (6).

## 2 Histopathological features

### 2.1 Morphologic features of GIST

**Figure 1:**
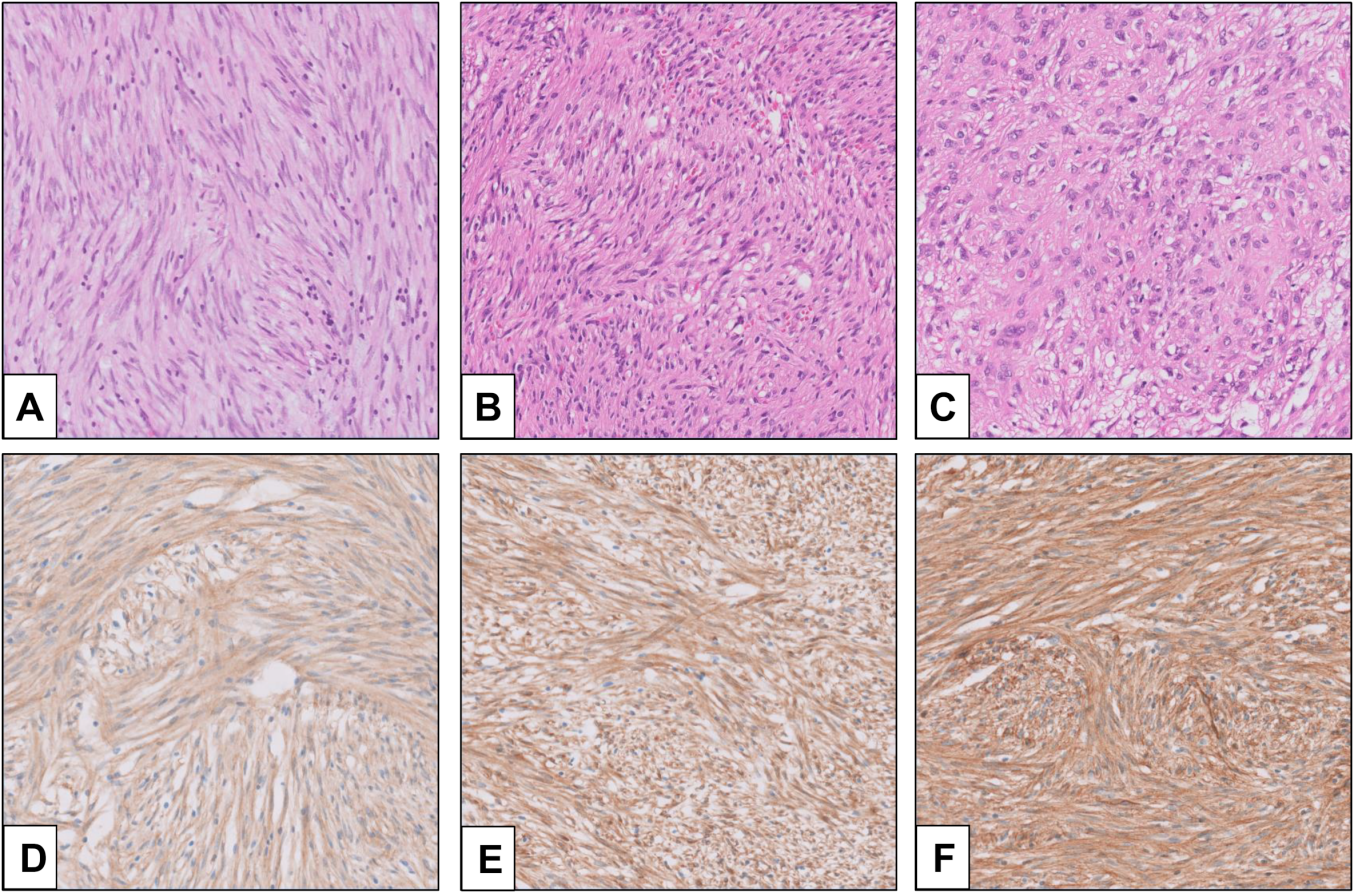
Morphology and immunohistochemistry (IHC) of GIST (provided by Seoul St. Mary’s Hospital). **(A)** Spindle cell-type GIST; **(B)** GIST with paranuclear vacuoles; **(C)** epithelioid-type GIST; **(D)** KIT IHC (rabbit polyclonal, Dako, Glostrup, Denmark, No. A4502, 1:400); **(E)** DOG1 IHC (mouse monoclonal, Novocastra, Newcastle, UK, K9, 1:200); and **(F)** CD34 IHC (mouse monoclonal, Agilent, Santa Clara, CA, USA, QBEnd-10, 1:100).

Histopathologically, three morphologic types are identified in GIST (7). The most common is the spindle type, which comprises 70% of the overall GIST (**Figure 1A**). In this type, bland spindle-shaped cells exhibit slightly eosinophilic cytoplasm arranged in a syncytial formation, with elongated nuclei and barely noticeable nucleoli (8). Artifactual paranuclear vacuoles are typical in stomach GIST (**Figure 1B**). Its subtypes include sclerosing, palisaded, vacuolated, diffuse hypercellular, or sarcomatoid features, marked by considerable nuclear irregularity and mitotic figures. Epithelioid GISTs, which constitute 20% of GISTs, consist of round cells featuring cytoplasm ranging from clear to eosinophilic arranged in clusters or nests (**Figure 1C**) (7). Their subtypes include sclerosing, discohesive, hypercellular, and sarcomatous tumors with significant atypia and mitotic activity. Mixed GISTs contain both spindle and epithelioid tumor cells. These comprise 10% of all GISTs. Some molecular phenotypes are associated with specific histology. SDH-deficient GISTs typically present with epithelioid or mixed tumor cells, multinodular pattern, minimal nuclear pleomorphism, and occasional atypical mitoses (6). In dedifferentiated GISTs, anaplastic cells with an unusual phenotype are observed (9). Unlike pleomorphic GISTs, dedifferentiated GISTs may lose expression of KIT and may exhibit aberrant expression of other markers, such as cytokeratin (10). The anatomical location of GISTs was not associated with the distribution of histological features (11).

### 2.2 Immunohistochemical features of GIST

Among *“*classic*”* GISTs, 95% show strong and diffuse expression of KIT (CD117) on immunohistochemistry (**Figure 1D**) (12). The intracellular staining is observed in cytoplasmic, membrane-associated, or perinuclear dot-like patterns. In a small subset, expression of KIT may be lacking or show very limited staining, especially in GISTs with PDGFRA mutations (13). In recent years, the chloride-channel protein ANO1/DOG1 has been recognized as an equally sensitive and specific marker (**Figure 1E**) (14). ANO1/DOG1 has been known to be positive in as many as 50% of KIT-negative GISTs (13). Another marker, CD34, is mainly expressed in spindle cell GISTs (especially gastric tumors), while epithelioid GISTs are less consistently positive (**Figure 1F**) (15). SDH-deficient GISTs exhibit loss of SDHB protein expression, regardless of which SDH gene (SDHA, SDHB, SDHC, and SDHD) is mutated (16). Loss of expression in neurofibromin (NF1) may help identify NF1-associated GISTs (15).

### 2.3 Histopathological differential diagnosis

Multiple tumors should be differentiated before GIST is diagnosed. For spindle-type GISTs, other spindle cell neoplasms, including leiomyoma, leiomyosarcoma, schwannoma, desmoid fibromatosis, solitary fibrous tumor, inflammatory myofibroblastic tumor, dedifferentiated liposarcoma, and inflammatory fibroid polyp, should be considered (17). Due to overlap in histology, other diagnostic modalities such as immunohistochemistry, fluorescence *in situ* hybridization (FISH), and next-generation sequencing should be accommodated (18). In epithelioid or mixed-type GISTs, perivascular epithelioid tumors (PEComa), metastatic melanoma, glomus tumors, and neuroendocrine neoplasms should be differentiated (18). These tumors are more challenging to diagnose because epithelioid and mixed-type GISTs exhibit occasional loss of expression for KIT and DOG1. Additional ancillary tests can be helpful in the differential diagnosis of these subtypes. In a much rarer histologic phenotype, extraskeletal myxoid chondrosarcoma and myoepithelial tumors of soft tissue could be considered when diagnosing GISTs with chondroid differentiation (19).

## 3 Prognosis

The Armed Forces Institute of Pathology (AFIP) classification and the Modified National Institutes of Health (NIH) classification are still the most commonly used in clinical practice for risk stratification of GISTs (20). The modified NIH criteria comprise four components: size, mitotic count, site, and presence of rupture (21). Any GISTs with a ruptured tumor should be categorized as high risk. Gastric GISTs show a better prognosis than extragastric GISTs, especially those smaller than 5 cm in size, and have a low recurrence rate (22, 23). In small gastric GISTs, a high mitotic index, aberrant p53 expression, and KIT exon 11 mutations are related to frequent recurrence (24). Other indicators associated with prognosis are gastrointestinal hemorrhage, high Ki67 index, nutritional index, tumor necrosis, and younger age (25).

## 4 Molecular classification

### 4.1 Overview of molecular features

Several key signaling pathways in GIST have been established (**Table 1**). KIT, PDGFRA, NF1, and SDH mutations are considered driver mutations (26, 27). KIT or PDGFRA mutations initiate the relevant signaling pathways, which constitutively activate MAPK, PI3K, and STAT3 signaling cascades (28, 29). Some GISTs harbor mutations in the RAS-family gene NF1 or BRAF (30, 31). These mutations mediate the downstream activation of the MAPK pathway. The NF1 protein regulates the RAS signaling pathway inactivating Guanosine diphosphate (GDP) by catalytically activating Guanosine triphosphate (GTP) hydrolysis (32). SDH-deficient GISTs exhibit the inactivation of the SDH complex within the mitochondria, leading to increased levels of hypoxia-inducible factor 1-α (HIF1α) and stimulating transcription of vascular endothelial growth factor (VEGF) and Insulin Like Growth Factor 2 (IGF2), which acts on the PI3K/AKT signaling and RAS/RAF/MAPK pathways (33).

**Table 1:** Molecular classification of GIST.

Gene	Mutation or location	Therapeutic options
KIT (60%–70%)	Exon 9, exon 11	Imatinib
	Exon 13, exon 14	Sunitinib, regorafenib, ripretinib
	Exon 17	Regorafenib, ripretinib
PDGFRA (10%–15%)	Exons 12, 14, and 18 (non-D842V)	Imatinib
	Exon 15	Resistance to TKIs
	Exon 18 (D842V)	Resistance to TKIs
KIT or PDGFRA wildtype		
NF1	Small intestine	Insensitive to TKIs
BRAF		BRAF inhibitors
NTRK translocations		TRK inhibitors
SDH-deficiency	SHDA, SDHB, SDHC, or SDHD mutant	Resistance to TKIs
	SDHC hypermethylation	Resistance to TKIs

Following initiation, an early aberration includes the loss of 14q, which is found in up to 70% of cases, resulting in somatic homozygous inactivating mutations in the MYC-associated factor X (MAX) gene located on chromosome 14q (34). Inactivation of MAX leads to the inactivation of p16, which increases tumor proliferation in the early phase. GISTs classified as intermediate and high risk exhibit losses on 22q, 1p, 15q, 13q, and 9p (which includes CDKN2A and p16INK4A) and/or gains at 5q, 8q, 16q, and 20q (18). Transition to high-grade GIST may require additional inactivating mutations in p16, RB1, and TP53. Inactivation of dystrophin, encoded by the DMD gene on Xp21.1, facilitates metastatic potential in as many as 90% of metastatic GISTs (35).

### 4.2 KIT and PDGFRA mutations

Approximately 80% of GISTs harbor activating mutations in either the KIT or PDGFRA genes (36, 37). Most KIT mutations are somatic, with the most frequent mutations occurring in exon 11. Deletions affecting codons 557–558 of exon 11 have been reported in approximately 28% of all GISTs, and are associated with high-risk tumors that have a higher mitotic index (> 5/50 HPF) and larger (> 5 cm) tumor size (37). Tumors with this molecular signature are commonly found in both gastric and nongastric sites and typically develop in individuals under 60 years of age. Although the overall prevalence of exon 11 mutations in gastric and nongastric locations appears to vary across studies (37–40), mutations in exon 9, 13, and 17 are more common in the small bowel. Additionally, mutations in exons 9 and 13 are often associated with a more aggressive phenotype (39, 41).

PDGFRA-mutated GISTs account for 10%–15% of overall GISTs (42, 43). These GISTs are predominantly of the epithelioid type and typically originate in the stomach. Exon 18 mutations are the most frequently observed. The status of PDGFRA exon 18 mutations correlates with a highly favorable disease outcome compared to KIT mutations in exons 9 and 11. However, missense mutation p. D842V is considered imatinib-resistant (44). Other non-p.D842V mutations are considered sensitive to imatinib (42). Exon 14 mutations are less common and are associated with a better prognosis.

Secondary resistance to imatinib treatment can lead to tumor progression in one or more lesions 12 to 36 months after the initial treatment. The resistance is caused by nonrandomly distributed Single nucleotide variants (SNVs) affecting codons in the Adenosine triphosphate (ATP)-binding pocket (exons 13 and 14) and the kinase activation loop (exons 17 and 18) of KIT. The V654A mutation in KIT exon 13 is a prevalent secondary resistance mutation.

### 4.3 Other mutations

GISTs that are wildtype for KIT or PDGFRA differ from KIT and PDGFRA-mutant GISTs in their clinical and molecular behavior (29). In SDH-competent, KIT and PDGFRA wildtype GISTs, NF1-related GISTs are often multicentric and predominantly located in the small intestine, frequently exhibiting loss of heterozygosity at 14q and 22q—traits similar to those found in sporadic KIT- and PDGFRA-mutated GISTs (45). BRAF mutation appear to be mutually exclusive with KIT and PDGFRA mutations. BRAF-mutated GISTs affect both sexes equally, are commonly located in the small bowel, and exhibit spindle cell tumor cell histology with variable clinical behavior (45, 46). Currently, there are no established clinical or prognostic associations linked to the status of BRAF mutations. Despite the extreme rarity of GISTs harboring ETV6-NTRK3 fusions, potent NTRK inhibitors are now available (47).

### 4.4 SDH-deficient GIST

Alterations in SDH subunit genes are detected in 5%–10% of GISTs. In SDH-deficient GISTs, 60% harbor inactivating mutations (nearly always germline), and 40% harbor SDHC promoter methylation (epimutation), leading to SDH dysfunction (16, 48). Mutations are most frequently observed in SDHA (30%), followed by SDHB, SDHC, and SDHD (together accounting for 20%–30%). Approximately half of SHD-deficient GISTs are caused by hypermethylation (16). SDH-deficient GISTs usually occur in patients < 40 years of age, have a female prevalence, and are most commonly located in the stomach (with the antrum being the most frequent site and the lesser curvature the second most common) (6, 49). Tumors exhibit characteristic morphologic features, including epithelioid or mixed epithelioid/spindle cells, multinodular and plexiform growth patterns, the occurrence of multiple tumors, less atypical or pleomorphic nuclei, lymphovascular involvement, and lymph node metastasis. The clinical course of SDH-deficient GISTs is relatively indolent compared to SDH-wildtype GISTs, but it is not sensitive to imatinib. Syndromic SDH-deficient GISTs include the Carney triad (CT) and Carney–Stratakis syndrome (CSS) (16, 50, 51). CT is characterized by the synchronous or metachronous occurrence of at least three different tumor types: GIST, pulmonary chondroma, and paraganglioma. CT mainly affects females, and no hereditary patterns have been reported. Most cases of CT exhibit downregulation of SDH due to site-specific hypermethylation of the SDHC gene. A distinct dyad of familial multicenter paraganglioma and gastric multifocal GISTs characterizes CSS. Unlike CT, CSS follows an autosomal dominant inheritance pattern that affects both sexes during childhood and adolescence. In CSS, SDH deficiency is caused by inactivating germline mutations or large deletions in the SDHB, SDHC, or SDHD genes, which encode the B, C, or D subunits of the SDH enzyme.

## 5 Conclusions

The standard treatment of primary GIST is surgery (52, 53). However, relapse and progression to advanced stages are substantial concerns. For metastatic GIST, surgery has limited values (54). Tyrosine kinase inhibitor (TKI) imatinib is considered a first-line therapy. Second (sunitinib) and third-line (regorafenib) TKI therapies have been developed due to the common occurrence of imatinib resistance (55, 56). However, these TKIs may also face resistance and are primarily effective against GISTs with KIT mutations. This is epically devastating for pediatric GIST, which most frequently presents as KIT and PDGFRA wildtype (57). Therefore, other treatment options, including novel fourth-line TKI, radiotherapy, combination TKIs, antibody-drug conjugates, and immunotherapy, are being developed. Recently, immune checkpoints such as PD-L1, PD-1, and CTLA-4 have emerged as potential prognostic biomarkers for GIST. Preclinical and clinical data have also highlighted the relevance of the tumor microenvironment in GISTs, with possible implications for clinical practice in the near future (58, 59). In a meta-analysis of rectal GISTs, neoadjuvant chemotherapy showed favorable results compared with adjuvant therapy (60). Therefore, with advanced knowledge of pathological and molecular features, further studies should be conducted to determine the optimal treatment strategy for patients with GIST.

Overall, GISTs represent a diverse and complex group of neoplasms with distinct histopathological, molecular, and clinical characteristics that significantly impact treatment and patient outcomes. While surgical resection remains the cornerstone of treatment, advances in underlying genetics, notably in KIT and PDGFRA mutations, have been pivotal in developing targeted therapies, including TKIs, and improving prognostic assessment. However, significant challenges remain, including managing resistance to TKIs and identifying novel treatments for SDH-deficient, wildtype, and pediatric GISTs, which are less responsive to current therapies. Continued research on GIST pathology and molecular biology, along with the exploration of novel therapeutic options such as immunotherapy, is essential for refining treatment strategies and improving outcomes for all GIST patients.

## References

[1] Agaimy A Wunsch PH. Gastrointestinal stromal tumours: A regular origin in the muscularis propria, but an extremely diverse gross presentation. A review of 200 cases to critically re-evaluate the concept of so-called extra-gastrointestinal stromal tumours. Langenbecks Arch Surg. (2006) 391:322–9. doi: 10.1007/s00423-005-0005-5 16402273

[2] Miettinen M Lasota J. Gastrointestinal stromal tumors: pathology and prognosis at different sites. Semin Diagn Pathol. (2006) 23:70–83. doi: 10.1053/j.semdp.2006.09.001 17193820

[3] Casella C Villanacci V D’Adda F Codazzi M Salerni B. Primary extra-gastrointestinal stromal tumor of retroperitoneum. Clin Med Insights Oncol. (2012) 6:189–97. doi: 10.4137/CMO.S9180 PMC3342024 22563251

[4] Lin J Liao W Wang J Li W Tang X Li H. Primary extra-gastrointestinal stromal tumor of retroperitoneum: clinicopathologic characteristics and prognosis of six cases. Front Oncol. (2023) 13:1033598. doi: 10.3389/fonc.2023.1033598 36895492 PMC9990817

[5] Soreide K Sandvik OM Soreide JA Giljaca V Jureckova A Bulusu VR. Global epidemiology of gastrointestinal stromal tumours (Gist): A systematic review of population-based cohort studies. Cancer Epidemiol. (2016) 40:39–46. doi: 10.1016/j.canep.2015.10.031 26618334

[6] Ibrahim A Chopra S. Succinate dehydrogenase-deficient gastrointestinal stromal tumors. Arch Pathol Lab Med. (2020) 144:655–60. doi: 10.5858/arpa.2018-0370-RS 31169996

[7] Miettinen M Majidi M Lasota J. Pathology and diagnostic criteria of gastrointestinal stromal tumors (Gists): A review. Eur J Cancer. (2002) 38 Suppl 5:S39–51. doi: 10.1016/s0959-8049(02)80602-5 12528772

[8] Miettinen M Lasota J. Gastrointestinal stromal tumors–definition, clinical, histological, immunohistochemical, and molecular genetic features and differential diagnosis. Virchows Arch. (2001) 438:1–12. doi: 10.1007/s004280000338 11213830

[9] Choi JJ Sinada-Bottros L Maker AV Weisenberg E. Dedifferentiated gastrointestinal stromal tumor arising *de novo* from the small intestine. Pathol Res Pract. (2014) 210:264–6. doi: 10.1016/j.prp.2013.12.008 24484970

[10] Antonescu CR Romeo S Zhang L Nafa K Hornick JL Nielsen GP. Dedifferentiation in gastrointestinal stromal tumor to an anaplastic kit-negative phenotype: A diagnostic pitfall: morphologic and molecular characterization of 8 cases occurring either *de novo* or after imatinib therapy. Am J Surg Pathol. (2013) 37:385–92. doi: 10.1097/PAS.0b013e31826c1761 PMC3728887 23348204

[11] Hu S Alpert L Cates JMM Gonzalez RS Rare GRSG. Gastrointestinal stromal tumors (Gists) arising in uncommon locations: clinicopathologic features and risk assessment of esophageal, colonic, and appendiceal gists. Mod Pathol. (2022) 35:554–63. doi: 10.1038/s41379-021-00949-w 34702994

[12] Miettinen M Sobin LH Sarlomo-Rikala M. Immunohistochemical spectrum of gists at different sites and their differential diagnosis with a reference to cd117 (Kit). Mod Pathol. (2000) 13:1134–42. doi: 10.1038/modpathol.3880210 11048809

[13] Patil DT Rubin BP. Gastrointestinal stromal tumor: advances in diagnosis and management. Arch Pathol Lab Med. (2011) 135:1298–310. doi: 10.5858/arpa.2011-0022-RA 21970485

[14] Corless CL Fletcher JA Heinrich MC. Biology of gastrointestinal stromal tumors. J Clin Oncol. (2004) 22:3813–25. doi: 10.1200/JCO.2004.05.140 15365079

[15] Hirota S. Differential diagnosis of gastrointestinal stromal tumor by histopathology and immunohistochemistry. Transl Gastroenterol Hepatol. (2018) 3:27. doi: 10.21037/tgh.2018.04.01 29971258 PMC6002266

[16] Schipani A Nannini M Astolfi A Pantaleo MA. Sdha germline mutations in sdh-deficient gists: A current update. Genes (Basel). (2023) 14:646. doi: 10.3390/genes14030646 36980917 PMC10048394

[17] Ieni A Barresi V Reggiani Bonetti L Branca G Caruso RA Tuccari G. Cytohistological and immunohistochemical characteristics of spindle-shaped mesenchymal neoplasms occurring in the gastrointestinal tract. Scand J Gastroenterol. (2017) 52:291–9. doi: 10.1080/00365521.2016.1251607 27817254

[18] Brcic I Argyropoulos A Liegl-Atzwanger B. Update on molecular genetics of gastrointestinal stromal tumors. Diagnostics (Basel). (2021) 11:194. doi: 10.3390/diagnostics11020194 33525726 PMC7912114

[19] Pulcini G Villanacci V Rossi E Gheza F Cervi E Ferrari AB. Gastrointestinal stromal tumor with chondroid differentiation. Anticancer Res. (2009) 29:2761–5. 19596958

[20] Joensuu H Vehtari A Riihimaki J Nishida T Steigen SE Brabec P. Risk of recurrence of gastrointestinal stromal tumour after surgery: an analysis of pooled population-based cohorts. Lancet Oncol. (2012) 13:265–74. doi: 10.1016/S1470-2045(11)70299-6 22153892

[21] Chen T Xu L Ye L Qiu H Hu Y Liu H. A new nomogram for recurrence-free survival prediction of gastrointestinal stromal tumors: comparison with current risk classification methods. Eur J Surg Oncol. (2019) 45:1109–14. doi: 10.1016/j.ejso.2018.12.014 30594406

[22] Hassan I You YN Shyyan R Dozois EJ Smyrk TC Okuno SH. Surgically managed gastrointestinal stromal tumors: A comparative and prognostic analysis. Ann Surg Oncol. (2008) 15:52–9. doi: 10.1245/s10434-007-9633-z 18000711

[23] Joensuu H. Risk stratification of patients diagnosed with gastrointestinal stromal tumor. Hum Pathol. (2008) 39:1411–9. doi: 10.1016/j.humpath.2008.06.025 18774375

[24] Kim MY Park YS Choi KD Lee JH Choi KS Kim DH. Predictors of recurrence after resection of small gastric gastrointestinal stromal tumors of 5 cm or less. J Clin Gastroenterol. (2012) 46:130–7. doi: 10.1097/MCG.0b013e31821f8bf6 21617541

[25] Zhang H Liu Q. Prognostic indicators for gastrointestinal stromal tumors: A review. Transl Oncol. (2020) 13:100812. doi: 10.1016/j.tranon.2020.100812 32619820 PMC7327422

[26] Rubin BP Heinrich MC. Genotyping and immunohistochemistry of gastrointestinal stromal tumors: an update. Semin Diagn Pathol. (2015) 32:392–9. doi: 10.1053/j.semdp.2015.02.017 25766843

[27] Alkhuziem M Burgoyne AM Fanta PT Tang CM Sicklick JK. The call of “the wild”-type gist: it’s time for domestication. J Natl Compr Canc Netw. (2017) 15:551–4. doi: 10.6004/jnccn.2017.0057 PMC5549855 28476734

[28] Li K Cheng H Li Z Pang Y Jia X Xie F. Genetic progression in gastrointestinal stromal tumors: mechanisms and molecular interventions. Oncotarget. (2017) 8:60589–604. doi: 10.18632/oncotarget.16014 PMC5601165 28947997

[29] Wu CE Tzen CY Wang SY Yeh CN. Clinical diagnosis of gastrointestinal stromal tumor (Gist): from the molecular genetic point of view. Cancers (Basel). (2019) 11:679. doi: 10.3390/cancers11050679 31100836 PMC6563074

[30] Hostein I Faur N Primois C Boury F Denard J Emile JF. Braf mutation status in gastrointestinal stromal tumors. Am J Clin Pathol. (2010) 133:141–8. doi: 10.1309/AJCPPCKGA2QGBJ1R 20023270

[31] Gasparotto D Rossi S Polano M Tamborini E Lorenzetto E Sbaraglia M. Quadruple-negative gist is a sentinel for unrecognized neurofibromatosis type 1 syndrome. Clin Cancer Res. (2017) 23:273–82. doi: 10.1158/1078-0432.CCR-16-0152 27390349

[32] Ratner N Miller SJ. A rasopathy gene commonly mutated in cancer: the neurofibromatosis type 1 tumour suppressor. Nat Rev Cancer. (2015) 15:290–301. doi: 10.1038/nrc3911 25877329 PMC4822336

[33] Joensuu H Hohenberger P Corless CL. Gastrointestinal stromal tumour. Lancet. (2013) 382:973–83. doi: 10.1016/S0140-6736(13)60106-3 23623056

[34] Schaefer IM Wang Y Liang CW Bahri N Quattrone A Doyle L. Max inactivation is an early event in gist development that regulates P16 and cell proliferation. Nat Commun. (2017) 8:14674. doi: 10.1038/ncomms14674 28270683 PMC5344969

[35] Wang Y Marino-Enriquez A Bennett RR Zhu M Shen Y Eilers G. Dystrophin is a tumor suppressor in human cancers with myogenic programs. Nat Genet. (2014) 46:601–6. doi: 10.1038/ng.2974 PMC4225780 24793134

[36] Nishida T Hirota S Taniguchi M Hashimoto K Isozaki K Nakamura H. Familial gastrointestinal stromal tumours with germline mutation of the kit gene. Nat Genet. (1998) 19:323–4. doi: 10.1038/1209 9697690

[37] Wozniak A Rutkowski P Piskorz A Ciwoniuk M Osuch C Bylina E. Prognostic value of kit/pdgfra mutations in gastrointestinal stromal tumours (Gist): polish clinical gist registry experience. Ann Oncol. (2012) 23:353–60. doi: 10.1093/annonc/mdr127 21527588

[38] Liang L Li X Li D Liu P Nong L Dong Y. Mutational characteristics of gastrointestinal stromal tumors: A single-center analysis of 302 patients. Oncol Lett. (2021) 21:174. doi: 10.3892/ol.2021.12435 33552291 PMC7798044

[39] Antonescu CR Sommer G Sarran L Tschernyavsky SJ Riedel E Woodruff JM. Association of kit exon 9 mutations with nongastric primary site and aggressive behavior: kit mutation analysis and clinical correlates of 120 gastrointestinal stromal tumors. Clin Cancer Res. (2003) 9:3329–37. 12960119

[40] Eskitzis P Michou V Theoti R Antoniou A Tsavlis D Anestakis D. Unraveling gastric and small intestine gastrointestinal stromal tumors: A review of our current knowledge. Gastrointestinal Disord. (2024) 6:842–57. doi: 10.3390/gidisord6040059

[41] Lasota J Corless CL Heinrich MC Debiec-Rychter M Sciot R Wardelmann E. Clinicopathologic profile of gastrointestinal stromal tumors (Gists) with primary kit exon 13 or exon 17 mutations: A multicenter study on 54 cases. Mod Pathol. (2008) 21:476–84. doi: 10.1038/modpathol.2008.2 18246046

[42] Farag S Somaiah N Choi H Heeres B Wang WL van Boven H. Clinical characteristics and treatment outcome in a large multicentre observational cohort of pdgfra exon 18 mutated gastrointestinal stromal tumour patients. Eur J Cancer. (2017) 76:76–83. doi: 10.1016/j.ejca.2017.02.007 28284172

[43] Joensuu H Rutkowski P Nishida T Steigen SE Brabec P Plank L. Kit and pdgfra mutations and the risk of gi stromal tumor recurrence. J Clin Oncol. (2015) 33:634–42. doi: 10.1200/JCO.2014.57.4970 25605837

[44] Kunstlinger H Binot E Merkelbach-Bruse S Huss S Wardelmann E Buettner R. High-resolution melting analysis is a sensitive diagnostic tool to detect imatinib-resistant and imatinib-sensitive pdgfra exon 18 mutations in gastrointestinal stromal tumors. Hum Pathol. (2014) 45:573–82. doi: 10.1016/j.humpath.2013.10.025 24444465

[45] Wada R Arai H Kure S Peng WX Naito Z. Wild type” Gist: clinicopathological features and clinical practice. Pathol Int. (2016) 66:431–7. doi: 10.1111/pin.12431 27427238

[46] Torrence D Xie Z Zhang L Chi P Antonescu CR. Gastrointestinal stromal tumors with braf gene fusions. A report of two cases showing low or absent kit expression resulting in diagnostic pitfalls. Genes Chromosomes Cancer. (2021) 60:789–95. doi: 10.1002/gcc.22991 PMC8715869 34398495

[47] Cao Z Li J Sun L Xu Z Ke Y Shao B. Gists with ntrk gene fusions: A clinicopathological, immunophenotypic, and molecular study. Cancers (Basel). (2022) 15:105. doi: 10.3390/cancers15010105 36612101 PMC9817796

[48] Casey RT Ten Hoopen R Ochoa E Challis BG Whitworth J Smith PS. Sdhc epi-mutation testing in gastrointestinal stromal tumours and related tumours in clinical practice. Sci Rep. (2019) 9:10244. doi: 10.1038/s41598-019-46124-9 31308404 PMC6629852

[49] Miettinen M Lasota J. Succinate dehydrogenase deficient gastrointestinal stromal tumors (Gists) - a review. Int J Biochem Cell Biol. (2014) 53:514–9. doi: 10.1016/j.biocel.2014.05.033 PMC4112081 24886695

[50] Pitsava G Settas N Faucz FR Stratakis CA. Carney triad, carney-stratakis syndrome, 3pas and other tumors due to sdh deficiency. Front Endocrinol (Lausanne). (2021) 12:680609. doi: 10.3389/fendo.2021.680609 34012423 PMC8126684

[51] Recht HS Fishman EK. Carney-stratakis syndrome: A dyad of familial paraganglioma and gastrointestinal stromal tumor. Radiol Case Rep. (2020) 15:2071–5. doi: 10.1016/j.radcr.2020.08.002 PMC7481509 32944103

[52] Eisenberg BL Judson I. Surgery and imatinib in the management of gist: emerging approaches to adjuvant and neoadjuvant therapy. Ann Surg Oncol. (2004) 11:465–75. doi: 10.1245/ASO.2004.09.011 15123459

[53] DeMatteo RP Lewis JJ Leung D Mudan SS Woodruff JM Brennan MF. Two hundred gastrointestinal stromal tumors: recurrence patterns and prognostic factors for survival. Ann Surg. (2000) 231:51–8. doi: 10.1097/00000658-200001000-00008 PMC1420965 10636102

[54] Kelly CM Gutierrez Sainz L Chi P. The management of metastatic gist: current standard and investigational therapeutics. J Hematol Oncol. (2021) 14:2. doi: 10.1186/s13045-020-01026-6 33402214 PMC7786896

[55] Xiao X Yuan W Wang C Song H. A Systematic Review and Network Meta-Analysis of the Efficacy and Safety of Third-Line and over Third-Line Therapy after Imatinib and Tki Resistance in Advanced Gastrointestinal Stromal Tumor. Front Pharmacol. (2022) 13:978885. doi: 10.3389/fphar.2022.978885 36479203 PMC9720279

[56] Schvartsman G Wagner MJ Amini B Zobniw CM Trinh VA Barbo AG. Treatment patterns, efficacy and toxicity of regorafenib in gastrointestinal stromal tumour patients. Sci Rep. (2017) 7:9519. doi: 10.1038/s41598-017-09132-1 28842575 PMC5573380

[57] Andrzejewska M Czarny J Derwich K. Latest advances in the management of pediatric gastrointestinal stromal tumors. Cancers (Basel). (2022) 14:4989. doi: 10.3390/cancers14204989 36291774 PMC9599787

[58] Dimino A Brando C Algeri L Gristina V Pedone E Peri M. Exploring the dynamic crosstalk between the immune system and genetics in gastrointestinal stromal tumors. Cancers (Basel). (2022) 15:216. doi: 10.3390/cancers15010216 36612211 PMC9818806

[59] Pilco-Janeta DF Garcia-Valverde A Gomez-Peregrina D Serrano C. Emerging drugs for the treatment of gastrointestinal stromal tumors. Expert Opin Emerg Drugs. (2021) 26:53–62. doi: 10.1080/14728214.2021.1896704 33645383

[60] Khan SI O’Sullivan NJ Temperley HC Rausa E Mehigan BJ McCormick P. Gastrointestinal stromal tumours (Gist) of the rectum: A systematic review and meta-analysis. Curr Oncol. (2022) 30:416–29. doi: 10.3390/curroncol30010034 PMC9857930 36661683

